# The Repeated Administration of Resveratrol Has Measurable Effects on Circulating T-Cell Subsets in Humans

**DOI:** 10.1155/2017/6781872

**Published:** 2017-05-04

**Authors:** J. Luis Espinoza, Ly Quoc Trung, Pleiades T. Inaoka, Kayoko Yamada, Dao T. An, Shohei Mizuno, Shinji Nakao, Akiyoshi Takami

**Affiliations:** ^1^Department of Hematology and Oncology, Kanazawa University Graduate School of Medical Sciences, Kanazawa, Japan; ^2^Phuong Chau International Hospital, Can Tho, Vietnam; ^3^Department of Physical Therapy, School of Health Sciences, Kanazawa University, Kanazawa, Japan; ^4^Department of Internal Medicine, Division of Hematology, Aichi Medical University School of Medicine, Nagakute, Aichi, Japan

## Abstract

Preclinical studies have shown that resveratrol exerts immunomodulatory effects with potential clinical value in the amelioration of autoimmune disorders and cancer prevention; however, little is known about the in vivo effects of this naturally occurring polyphenol on human immune cells. We assessed the effects of repeated doses of resveratrol (1000 mg/day for 28 days) on circulating immune cells in healthy Japanese individuals. Resveratrol was safe and well tolerated and was associated with significant increases in the numbers of circulating *γδ* T cells and regulatory T cells and resulted in small, yet significant, decreases in the plasma levels of the proinflammatory cytokines TNF-*α* and MCP-1 and a significant increase in the plasma antioxidant activity compared with the corresponding antioxidant baseline activity and with that in four control individuals. In in vitro studies, resveratrol significantly improved the growth of *γδ* T cells and regulatory T cells. These findings demonstrate that resveratrol has some clear biological effects on human circulating immune cells. Further studies are necessary to interpret the long-term immunological changes associated with resveratrol treatment.

## 1. Introduction

Resveratrol is a naturally occurring polyphenolic stilbene that is produced by certain plants in response to various stress stimuli including, microbe infection, ultraviolet radiation, and injury [1]. As a plant-derived substance, resveratrol can be found in various dietary sources including grapes, berries, peanuts, and red wine. Another important source of resveratrol is the Japanese knotweed, which has long been used in Japan and China as a traditional herbal remedy [2]. Resveratrol is the most extensively studied stilbene, and preclinical studies have revealed that this compound has several biological effects including, antioxidant, anti-inflammatory, and cytotoxic effects against human cancer cell lines derived from various tumor types [1, 3, 4].

Data from clinical trials indicate that daily doses of resveratrol between 20 mg and 2 g are safe and well tolerated [5–8]. For example, in a randomized placebo-controlled double-blinded clinical trial, resveratrol, given at 1 g per day for a period of 45 days in patients with type 2 diabetes, was not associated with serious side effects [9]. In patients with Alzheimer's, resveratrol, given for one year, starting at 500 mg and going up to 2 g per day, was not associated with relevant secondary effects [10]. When given at doses higher than 2 g daily (maximum 5 g/day), resveratrol is well tolerated by healthy individuals, although mild to moderate gastrointestinal symptoms, including nausea, flatulence, abdominal discomfort, and diarrhea are frequently observed [11, 12].

Preclinical studies have shown that resveratrol induces immunomodulatory effects, including the upregulation of NKG2D receptors on natural killer cells (NK) that it promotes the NKG2D-dependent killing of tumor cells [13, 14] and the induction of regulatory T cells (Treg) in the colonic tissues of mice exposed to colonic inflammatory stress [15]. However, it is unknown whether resveratrol exerts measurable immunomodulatory effects in humans. Thus, the current study evaluated the effects of repeated oral doses of resveratrol on circulating immune cells in healthy individuals. The effects were assessed by collecting blood samples and analyzing the potential changes at baseline and every two weeks for a period of six weeks. Our data revealed that resveratrol induced measurable changes in circulating immune cells in humans and showed considerable antioxidant activities and other biological effects in humans in vivo.

## 2. Materials and Methods

### 2.1. Study Design

A total 9 healthy volunteers (male, *n* = 7; female, *n* = 2; median age, 36 years (range, 30–50 years); median body weight, 61.5 kg (range, 50–72 kg); median body length 171 cm (range, 158–184 cm); median body mass index (BMI), 20 kg/m^2^ (range, 19–27 kg/m^2^)) were enrolled in this phase 1 randomized trial (UMIN-CTR #UMIN000007690), which was approved by the Kanazawa University Hospital Institutional Review Board (#2011-046-5741) and conducted in accordance with the Declaration of Helsinki. All subjects provided their written informed consent to participate in this study. The inclusion criteria were as follows: the absence of subjective symptoms, a normal liver and renal function, drinking alcohol ≤3 times per week, and willingness to abstain from the ingestion of resveratrol-containing foods and drink. The exclusion criteria were as follows: the long-term use of medication, chronic disease, a history of invasive cancer within 5 years, a history of noninvasive cancer within one year, and a history of smoking within one year. To control potential spontaneous changes in immune cells that were not associated with the administration of resveratrol, the study included a group of four healthy subjects that followed the same instructions as the resveratrol group with regard to refraining from ingesting wine or resveratrol-rich foods and who were willing to provide blood samples every two weeks. The enrolled subjects were randomized 1 : 1 to ingest two capsules (500 mg each) of resveratrol daily (Transmax, Biotivia, WI, USA) consisting of 100% resveratrol (five subjects); the control group (*n* = 4) received no treatment. The subjects in the resveratrol group were instructed to ingest resveratrol after lunch daily for 28 days and were evaluated on a weekly basis to investigate their compliance and the incidence of adverse events according to the National Cancer Institute Common Terminology Criteria for Adverse Events (CTCAE) version 4.0 [16]. The primary objective of this trial was to evaluate the safety of resveratrol in Japanese individuals, and the secondary objective included assessing the potential effects of resveratrol on circulating lymphocytes and on serological parameters of oxidative stress.

### 2.2. Sample Preparation and Pharmacokinetic Evaluations

Blood samples were collected in 7 mL heparinized collection tubes at baseline and at days 14, 21, and 28 after study entry. From each sample, 2 mL of blood was centrifuged under refrigeration (10 min, 1300 ×g) to isolate plasma. To prevent potential light damage of resveratrol and its metabolites, the plasma was transferred to 1.5 mL LightSafe centrifuge tubes (Sigma-Aldrich, St. Louis, MO, USA) and stored at −70°C until use.

Peripheral blood mononuclear cells (PBMCs) were isolated from the remaining 5 mL of the blood using gradient centrifugation. A fraction of the isolated PBMCs from each sample was immediately analyzed by flow cytometry to assess the lymphocyte populations and the expression of relevant surface markers; the other fraction was cryopreserved for further use. A high-performance liquid chromatography (HPLC) analysis for the quantification of resveratrol was performed as described in a previous study [17].

### 2.3. Plasma Antioxidant Capacity

The plasma antioxidant capacity was measured by a metmyoglobin assay using an Antioxidant Assay Kit (Cayman) as previously described [18]. The assay relies on the ability of plasma antioxidant substances to inhibit the oxidation of ABTS to the radical cation ABTS^+^ by metmyoglobin, and the capacity of the antioxidants in the sample to prevent ABTS oxidation is compared with that of the tocopherol analogue Trolox and quantified as molar Trolox equivalents. The measurements were performed in samples collected at baseline and at two and four weeks after consumption.

### 2.4. The Measurement of Urinary 8-Oxo-2′-deoxyguanosine (8-OHdG)

Urine samples were collected from the participants in sterile tubes at baseline and after 2 weeks of resveratrol administration, and the urinary levels of 8-OHdG, adjusted by the creatinine concentration correction method, were performed at the Japan Institute for the Control of Aging (JaICA), Nikken Sail Co., Ltd. (Fukuroi, Shizuoka, Japan).

### 2.5. Flow Cytometry

Freshly isolated PBMCs were stained with antibodies specific to the cell surface markers of T cells, B cells, and the NK lymphocyte lineage, including anti-CD3, CD16, anti-CD19, CD56, CD4, CD8, *γδ* TCR (BD Biosciences, San Jose, CA, USA), anti-NKG2D, and anti-DNAM-1 (R&D Systems, Minneapolis, MN, USA). The cells were also stained with NIR zombie dye (BioLegend, San Diego, CA, USA) to exclude dead cells from the analysis. The proportion of circulating regulatory T cells (cTreg) was assessed in cryopreserved PBMCs. The stained cells were analyzed using a BD Canto instrument (BD Biosciences), and the data were analyzed using the FlowJo software package (version 10.1, Tree Star, Ashland, OR, USA).

### 2.6. The Plasma Cytokine Analysis

The measurement of plasma cytokines was performed using a MILLIPLEX^®^ MAP human cytokine immunoassay, Catalog# MPXHCYTO-60K-06, in accordance with the manufacturer's instructions (Millipore). The following cytokines and growth factors were tested: monocyte chemoattractant protein (MCP-1), chemokine C-X-C-motif chemokine 10 (CXCL10), tumor necrosis factor alpha (TNF-*α*), IL-1 receptor antagonist (IL-1ra), FMS-like tyrosine kinase-3 ligand (FLT3L), and vascular endothelial growth factor (VEGF). The assay sensitivity ranged from 0.1 to 23.4 pg/mL, depending on the analyte.

### 2.7. Cell Culture

To evaluate the effects of resveratrol on the ex vivo expansion of human *γδ* T cells, PBMCs from healthy individuals were seeded onto 12-well plates (1 × 10^6^ cells/mL) and cultured for seven days in Optimizer CST medium (Gibco-Thermo Scientific) supplemented with 100 IU/mL of recombinant IL-2, 10 ng/mL of recombinant IL-15 (PeproTech, Rocky Hill, NJ, USA), and 10% FBS (referred hereafter referred to as T-cell medium), in the presence of E-4-hydroxy-3-methyl-but-2-enyl pyrophosphate (HMBPP) and several concentrations of resveratrol (Sigma-Aldrich) or vehicle (0.5% DMSO). The cells were then stained with fluorochrome-labeled anti-CD3, anti- *γδ* TCR, anti-NKG2D, and anti-CD197 antibodies and analyzed by flow cytometry. The ex vivo growth of regulatory T cells (Treg) was assessed by culturing PBMCs in T-cell medium supplemented with anti-CD3/CD28 magnetic beads (Invitrogen, Waltham, MA, USA) for seven days in the presence or absence of several concentrations of resveratrol or vehicle. The cells were stained with fluorochrome-labeled anti-CD3, anti-CD25, anti-CD4, and anti-CD127 antibodies and analyzed by flow cytometry and were further confirmed as classical Treg cells by staining them with anti-CD3, anti-CD4, and anti FoxP3 antibodies, which was performed using a Foxp3 staining kit (eBiosciences).

### 2.8. Statistical Analysis

All of the data are reported as the mean ± standard deviation. Since the levels of most of the factors that were assessed—including the percentage of cells and cytokines—showed spontaneous and variable changes during the course of the study (even in the control group), to properly analyze the impact of resveratrol intervention on any given factor, the comparisons in this study were based on the fold change, which was calculated as the ratio of difference between the final value (value at a given time) and the initial value (baseline level) over the initial value. Thus, if the baseline value was *A* and final value was *B*, the fold change was (*B* − *A*)/*A*. When comparisons were made between two different groups, the statistical significance was determined using Student's *t*-test. The statistical significance of multiple comparisons was determined using a one-way analysis of variance. *P* values of ≤0.05 were considered to indicate statistical significance. The analyses were performed and graphs were drawn using the GraphPad Prism software package.

## 3. Results

### 3.1. The Safety and Pharmacokinetic Evaluations and Plasma Antioxidant Studies

**Figure 1:**
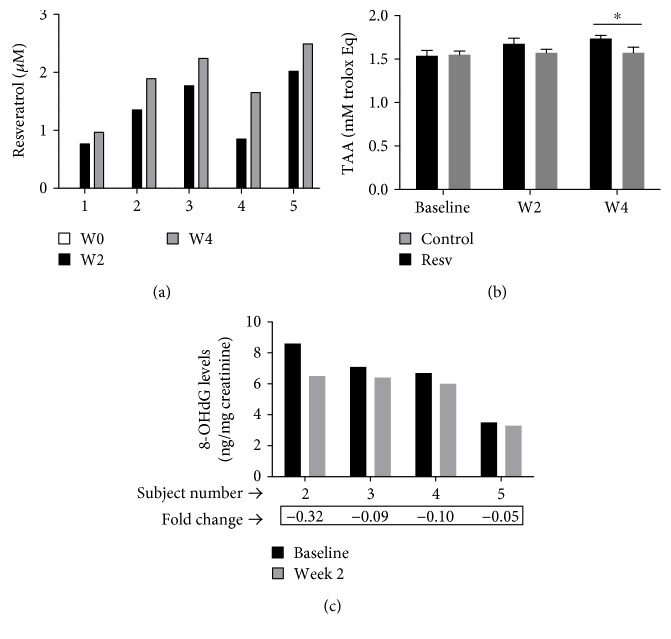
Pharmacokinetic studies and the parameters of plasma oxidative stress. (a) The plasma levels of resveratrol in samples collected at baseline and after two and four weeks of resveratrol consumption. (b) The total antioxidant activity (TAA) in plasma from healthy individuals measured at baseline and at after two (W2) and four weeks (W4) of study entry. Data is expressed as mean ± standard error. ∗ indicates statistical significance. (c) The urinary 8-OHdG levels, adjusted by the creatinine concentration correction method, in four individuals at baseline and after two weeks of resveratrol administration.

All five subjects enrolled in this study received two capsules of resveratrol daily (1 g/day) for 28 consecutive days. Among the participants in the resveratrol group, two subjects experienced mild nausea and flatulence that resolved without sequelae or intervention. No other adverse events were reported. The plasma resveratrol concentrations measured at after two and four weeks of resveratrol administration ranged from 0.7 to 2.55 *μ*M and were in agreement with average plasma concentration (*C*_av_) of resveratrol reported in previous studies [19]. We did not investigate the compound concentration that reach the maximum plasma level (*C*_max_) and the area under the curve (AUC) or perform other detailed pharmacokinetic analyses in the present study because the pharmacokinetics of resveratrol have been extensively studied in humans [19–21]. As expected, resveratrol was not detected in any of the plasma samples collected at baseline or in the samples derived from the control group (Figure 1(a)). As shown in Figure 1(b), the administration of resveratrol was associated with increases in the overall antioxidant capacity in the plasma samples in comparison to samples collected at baseline and samples from the control group, and this effect was more noticeable after four weeks of resveratrol administration (1.673 ± 0.09) versus that of control (1.538 ± 0.07). Furthermore, the 8-OHdG levels in urine samples collected at 2 weeks after study entry were found to have decreased in comparison to the baseline levels in subjects in the resveratrol group (Figure 1(c)). The levels of 8-OHdG in the samples from the control group and from one subject from the resveratrol group were not available for the analysis; thus, the clinical significance of this finding is unknown.

### 3.2. The Effects of Resveratrol on Circulating Immune Cells

**Figure 2:**
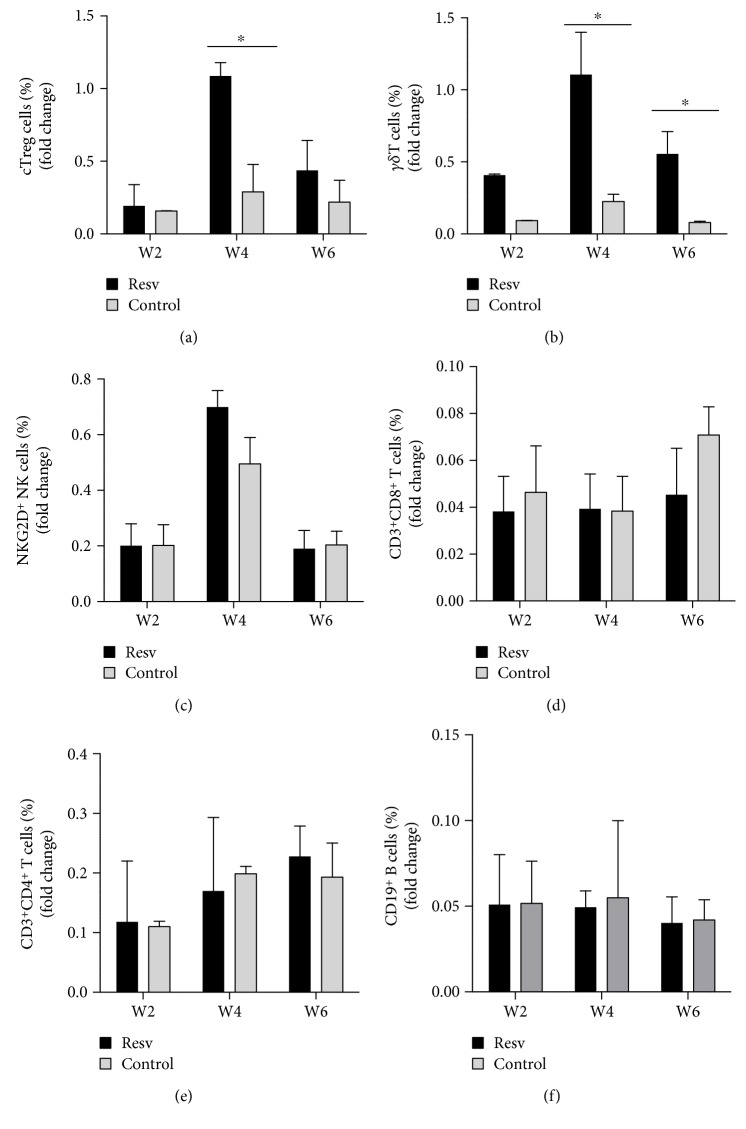
The effects of resveratrol on circulating immune cells (a) PBMCs from blood samples collected at baseline or at the indicated time (weeks) were analyzed by flow cytometry. The percentage of CD3^+^CD4^+^CD25^+^CD127^−^ circulating Treg cells (a), CD3^+^NKG2D^+^*γδ* T cells (b), CD3^+^CD56^+^NKG2D^+^ NK cells (c), CD3^+^CD8^+^ T cells (d), CD3^+^CD4^+^ T cells (e), and CD19^+^ B cells (f) are shown. The figures indicate the mean ± SD fold change at the indicated time point. ^∗^*p* < 0.05.

Flow cytometry of PBMCs from blood samples collected at baseline and every two weeks revealed a statistically significant increase in the percentage of circulating Treg cells (cTreg), defined as CD3^+^, CD4^+^, CD25^+^, and CD127^dim/neg^ lymphocytes [22], in association with the administration of resveratrol (Figure 2(a)). In addition, after two weeks of resveratrol administration, the number of *γδ*^+^ NKG2D^+^ T cells was found to be significantly increased in all five individuals in comparison to the baseline levels and the levels in the subjects of the control group (Figure 2(b)). We observed a tendency toward an increase in the percentage of CD3^−^, CD56^+^ NKG2D^+^NK cells at four weeks after study entry in the resveratrol group; however, it was not statistically significant (Figure 2(c)). Conversely, the percentage of CD8^+^ T cells (Figure 2(d)), CD4^+^ T cells (Figure 2(e)), and CD19^+^ B cells (Figure 2(f)) remained essentially unchanged throughout the course of the study.

### 3.3. The Effects of Resveratrol on Plasma Cytokines

**Figure 3:**
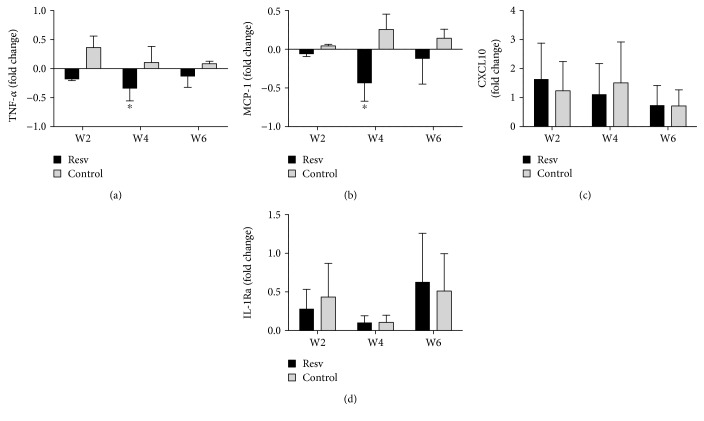
The plasma expression of relevant cytokines and chemokines. The cytokine levels of plasma samples collected at 2, 4, and 6 weeks after study entry were measured using a multiplex cytokine assay. The fold changes of TNF-*α* (a), MCP-1 (b), CXCL10 (c), and IL-1Ra (d) are shown. ^∗^*p* < 0.05.

To better ascertain the biological effects of resveratrol on immune cells, we simultaneously measured the various immune response-associated cytokines in plasma samples by a multianalyte assay. In comparison to that in the control group, the plasma levels of the proinflammatory cytokines TNF-*α* (Figure 3(a)) and MCP-1 (Figure 3(b)) in the resveratrol group showed modest but significant decreases. These changes were noticeable within two weeks after study entry and were still present in samples collected at week six. The levels of other cytokines, including CXCL-10 (Figure 3(c)) and IL-1Ra (Figure 3(d)), remained essentially unchanged. VEGF and FLT3L were not consistently detectable in the samples tested and were therefore not included in the analysis.

### 3.4. Resveratrol Promotes the Ex Vivo Expansion of *γδ* T Cells

**Figure 4:**
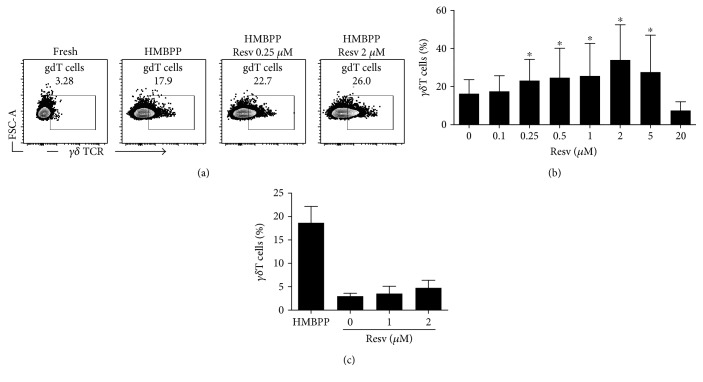
The effects of resveratrol on the in vitro proliferation of human *γδ* T cells. PBMCs from healthy donors were cultured for seven days in T-cell medium stimulated with HMBPP in the presence of the indicated concentrations of resveratrol. The percentage of CD3^+^*γδ* T cells was determined by flow cytometry. (a) A representative flow cytometry result showing cells cultured with HMBPP alone (control) or with HMBPP together with resveratrol (resv). (b) A summary of the flow cytometry data showing the percentage of *γδ* T cells derived from three healthy individuals. (c) PBMCs from two healthy donors were cultured with HMBPP as a positive control or with various concentrations of resveratrol (resv) but without HMBPP. The percentage of *γδ* T cells was assessed in the same manner as (a). ^∗^*p* < 0.05.

To gain further insight into the biological effects of resveratrol on immune cells, we first tested the in vitro proliferation of *γδ* T cells from IL-2/IL-15 stimulated PBMCs derived from healthy individuals in the presence of various concentrations of resveratrol in culture medium supplemented with HMBPP, a natural phosphoantigen produced by many bacteria and a potent activator of human *γδ* T cells [23]. A significant increase in the percentage of *γδ* T cells (>3 times the background of nonstimulated cells) was observed in response to HMBPP stimulation in all of the samples tested. Notably, when resveratrol was added to the cultures, at concentrations that ranged from 0.25 *μ*M to 5 *μ*M, *γδ* T cells proliferated more efficiently in comparison to cultures without resveratrol. The maximum *γδ* T cell proliferation was observed when the concentration of resveratrol in the culture medium was 2 *μ*M (Figures 4(a) and 4(b)). Of note, in the absence of specific stimuli to trigger *γδ* T cell proliferation, the number of these cells did not increase significantly, regardless of the presence or the absence of resveratrol in the culture medium (Figure 4(c)). This result suggests that rather than directly inducing *γδ* T cell proliferation, this agent may act as cofactor favoring *γδ* T cells, when cell growth is induced by an appropriate triggering signal.

### 3.5. Resveratrol Promotes the Proliferation of Regulatory T Cells In Vitro

**Figure 5:**
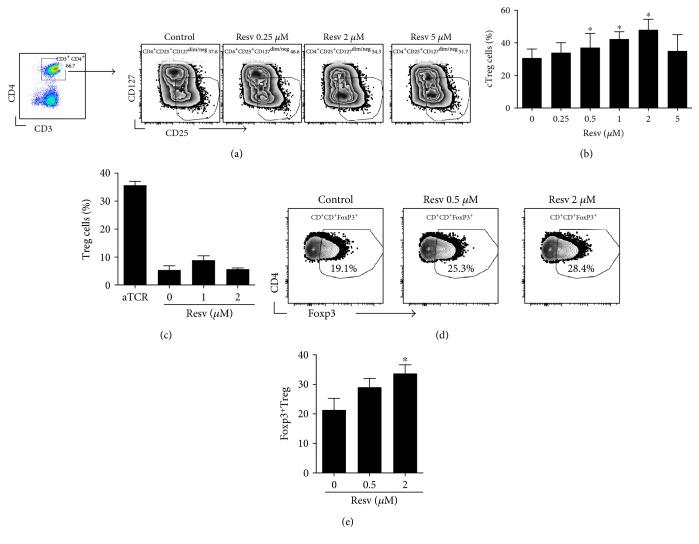
The effects of resveratrol on the in vitro proliferation of human Treg cells. PBMCs from healthy donors were cultured for seven days in T-cell medium stimulated with anti-CD3/CD28 beads to activate TCR in the presence of the indicated concentrations of resveratrol. (a) A representative flow cytometry result showing the percentage of CD3^+^CD4^+^CD25^+^CD127^−^ cTreg cells. (b) A summary of the flow cytometry data showing the percentage of CD3^+^CD4^+^CD25^+^CD127^−^ cTreg cells derived from three healthy individuals. (c) PBMCs from two healthy donors were cultured with anti-CD3/CD28 beads as positive control or with various concentrations of resveratrol (resv) but without anti-CD3/CD28 beads. The percentage of CD3^+^CD4^+^CD25^+^CD127^−^ cTreg cells was assessed in the same manner as (a). (d) PBMCs from three healthy donors were cultured for seven days in T-cell medium stimulated with anti-CD3/CD28 beads in the presence of the indicated concentrations of resveratrol. Flow cytometry analysis was performed to enumerate the percentage of Treg cells, which were gated as live^+^CD3^+^CD4^+^Foxp3^+^Treg cells. A representative flow cytometry result is shown. (e) A summary of the flow cytometry data showing the percentage of CD3^+^CD4^+^Foxp3^+^Treg cells derived from three healthy individuals. ^∗^*p* < 0.05.

We next assessed the growth of cTreg cells from PBMCs of healthy individuals that were cultured under IL-2/IL-15 and T-cell receptor (TCR) stimulation in the presence or absence of resveratrol. Interestingly, cTreg cells expanded from 4% to 36% in medium without resveratrol and by up to 47% in culture medium containing 2 *μ*M resveratrol (Figures 5(a) and 5(b)). Importantly, the numbers of in vitro expanded CD3^+^ CD4^+^ Foxp3^+^ cells, representing classical or conventional Treg cells [24], were also significantly higher when resveratrol was added to the culture medium, with higher numbers in medium containing 2 *μ*M resveratrol (Figure 5(c)). In the absence of TCR stimulation, however, resveratrol failed to increase the numbers of Treg cells from PBMCs (Figure 5(d)), thus indicating that resveratrol may only favor the growth of Treg cells when specific conditions to induce the growth of these cells are achieved. It must be noted that the concentrations of resveratrol that effectively improved the growth of *γδ* T cells and Treg cells in vitro were similar to the plasma resveratrol levels achieved in vivo—thus substantiating the biological and clinical relevance of these findings.

## 4. Discussion

Data from preclinical studies have attracted considerable attention on the potential health benefits of resveratrol. Various clinical trials have been conducted to explore the impact of resveratrol on obesity, diabetes, cardiovascular disease, and other age-associated diseases [5, 25]. Although the data from some of these trials appears to be consistent with that of the results from preclinical studies, conflicting and even negative results have also emerged, particularly from trials in which the potential health-promoting effects of resveratrol were evaluated in healthy or asymptomatic individuals [5, 8]. The limitations of the study design and the lack of appropriate surrogate markers of positive responses in studies investigating the effects of resveratrol as a primary prevention agent in nonclinical populations appear to explain the discrepancies among these studies, since defining clinical improvement in healthy individuals is a challenging task [19, 25]. In the present study, resveratrol (1000 mg, daily) for 28 consecutive days was well tolerated by healthy Japanese individuals and resulted in relevant biological effects on circulating immune cells that may have health promoting potential.

Resveratrol was associated with an increased number of circulating *γδ* T cells expressing the NKG2D receptor—a finding that was validated by in vitro studies showing that *γδ* T cells proliferated more efficiently when resveratrol was present in the culture medium. *γδ* T cells constitute a heterogeneous T-cell subset with important roles in tumor surveillance, as demonstrated by their protective role during tumor development in various mouse cancer models [23, 26]. *γδ* T cells express TCR and activate NK receptors, such as NKG2D, which is a stimulatory receptor that plays a crucial role in immunosurveillance by promoting the killing of transformed cells expressing specific stress-induced ligands [27, 28]. Accordingly, it is hypothesized that the tumor chemopreventive effects of resveratrol may involve the potentiation of tumor immunosurveillance by promoting the growth of NKG2D^+^ cells, such as *γδ* T cells.

The other relevant finding of this study is that the administration of resveratrol was associated with small but significant increases in the proportion of cTreg cells. This was further corroborated by in vitro experiments, which demonstrated the more efficient proliferation of Treg cells when TCR-activated PBMCs were cultured in the presence of resveratrol. Since resveratrol does not act as a direct inducer of Treg or *γδ* T cells, it is conceivable that this compound may trigger the cellular events that favor the growth of these cells. These effects may be mechanistically related to the inhibitory effect of resveratrol on the mTOR signal pathway, since resveratrol induces apoptosis via the inhibition of mTOR [29]. Interestingly the suppression of the mTOR signal with rapamycin promoted the generation of FoxP3^+^ Treg cells in vitro, despite the presence of Th17 polarizing cytokines [30] and the genetic ablation of mTOR in T cells resulted in the more efficient generation of Treg cells upon activation but not Th1 or Th2 cells [31], which are consistent with the in vitro findings of the present study. Moreover, in a mouse model of chemically induced colitis, the increased numbers of Treg cells and the concomitant suppression of mTOR signaling were reported in the intestinal tissue of mice treated with resveratrol [15].

Alternatively, the generation of reactive oxygen species (ROS) may be implicated, at least in vitro, in the favorable growth of Treg cells in the presence of resveratrol—since ROS-mediated apoptosis has been linked to resveratrol cytotoxicity [32]. Notably, although the sensitivity of T cells to ROS varies among different subsets [33], T lymphocytes themselves are a main source of ROS, particularly during TCR activation, and ROS are capable of inducing apoptosis in T cells; thus, while conventional T cells are highly sensitive to ROS-induced apoptosis, Treg cells have lower intracellular ROS levels and are protected from H_2_O_2_-induced death [34].

The clinical value of our finding that the administration of resveratrol was associated with small yet statistically significant decreases in the plasma levels of MCP-1 and TNF-*α* in healthy individuals remains unknown; however, given the involvement of these cytokines in inflammation, this finding may be consistent with the hypothesis that resveratrol possesses anti-inflammatory properties. Interestingly, decreases in the plasma levels of TNF-*α* and IL-6 were reported in healthy athletes after six weeks of resveratrol treatment [35]. In this regard, the plasma levels of MCP-1, IL-6, and TNF-*α* could be used as surrogate biomarkers in clinical trials evaluating autoimmune disorders that are associated with elevated levels of these cytokines, such as rheumatoid arthritis and psoriasis [36, 37]. Our observation that resveratrol favored the growth of Treg cells, a subset of T cells with fundamental roles in inflammation control, which are frequently diminished in patients with autoimmune disorders [38], together with the decreased plasma levels of MCP-1 and TNF-*α* is consistent with the findings of preclinical studies suggesting that resveratrol has anti-inflammatory properties [39].

In agreement with its polyphenol features, the consumption of resveratrol resulted in increased antioxidant activities in the plasma of the participating subjects. Oxidative stress has been implicated in the damage to biological macromolecules, including DNA, lipids, and proteins [40], and is proposed as one of the underlying molecular mechanisms of aging [41], atherosclerosis, inflammation, diabetes, and cancer [40, 42, 43]. In this study, the administration of resveratrol was associated with significantly decreased levels of 8-OHdG, another oxidative stress marker involved in DNA damage [44]. Interestingly, a high 8-OHdG level has been reported to be a risk factor for several cancers [45]. Thus, the finding that the repeated administration of resveratrol resulted in the improvement of markers of oxidative stress in vivo may be a potential mechanism by which this agent exerts its cancer chemopreventive effects in humans. Further studies should be performed to establish whether the administration of resveratrol for periods exceeding 28 days can persistently decrease oxidative stress markers—in particular the levels of 8-OHdG. In addition, the testing of these effects in individuals who are known to have increased levels of 8-OHdG, such as heavy smokers or individuals with metabolic syndrome, may be considered as surrogate biomarkers for evaluating interventional responses in clinical trials.

The study population was an important limitation of the present study. The inclusion of a control group helped us to elucidate the changes in the circulating immune cells that were most likely to have been induced by the administration of resveratrol. In addition, the inclusion of in vitro experiments to validate the effects of resveratrol on circulating immune cells under controlled culture conditions helped to strengthen the in vivo findings; however, given the limited sample size, the associations that were found should be considered part of a hypothesis-generating study. Future studies involving a greater number of participants and longer exposure to resveratrol will be necessary to confirm these observations and elucidate the mechanisms involved.
